# Neutralizing blood-borne polyphosphate *in vivo* provides safe thromboprotection

**DOI:** 10.1038/ncomms12616

**Published:** 2016-09-06

**Authors:** Linda Labberton, Ellinor Kenne, Andy T. Long, Katrin F. Nickel, Antonio Di Gennaro, Rachel A. Rigg, James S. Hernandez, Lynn Butler, Coen Maas, Evi X. Stavrou, Thomas Renné

**Affiliations:** 1 Institute of Clinical Chemistry and Laboratory Medicine, University Medical Center Hamburg-Eppendorf, Martinistrasse 52, 20246 Hamburg, Germany; 2 Clinical Chemistry, Department of Molecular Medicine and Surgery, L1:00, Karolinska Institutet and University Hospital, 17176 Stockholm, Sweden; 3 Department of Biomedical Engineering, School of Medicine, Oregon Health & Science University, 3303 SW Bond Avenue, Portland, Oregon 97239, USA; 4 Division of Laboratory Medicine, Mayo Clinic in Arizona, 13400 East Shea Boulevard, Scottsdale, Arizona 85259, USA; 5 Department of Clinical Chemistry and Haematology, University Medical Center Utrecht, Heidelberglaan 100, 3584 CX, Utrecht, The Netherlands; 6 Department of Medicine, Louis Stokes Veterans Administration Hospital, 10701 East Boulevard, Cleveland, Ohio 44106, USA; 7 Division of Hematology and Oncology, Department of Medicine, Case Western Reserve University, 10900 Euclid Avenue, Cleveland, Ohio 44106, USA

## Abstract

Polyphosphate is an inorganic procoagulant polymer. Here we develop specific inhibitors of polyphosphate and show that this strategy confers thromboprotection in a factor XII-dependent manner. Recombinant *Escherichia coli* exopolyphosphatase (PPX) specifically degrades polyphosphate, while a PPX variant lacking domains 1 and 2 (PPX_Δ12) binds to the polymer without degrading it. Both PPX and PPX_Δ12 interfere with polyphosphate- but not tissue factor- or nucleic acid-driven thrombin formation. Targeting polyphosphate abolishes procoagulant platelet activity in a factor XII-dependent manner, reduces fibrin accumulation and impedes thrombus formation in blood under flow. PPX and PPX_Δ12 infusions in wild-type mice interfere with arterial thrombosis and protect animals from activated platelet-induced venous thromboembolism without increasing bleeding from injury sites. In contrast, targeting polyphosphate does not provide additional protection from thrombosis in factor XII-deficient animals. Our data provide a proof-of-concept approach for combating thrombotic diseases without increased bleeding risk, indicating that polyphosphate drives thrombosis via factor XII.

## Abstract (web summary)

The inorganic procoagulant polymer polyphosphate participates in thrombosis via factor XII. Here the authors use recombinant probes that specifically bind or degrade circulating polyphosphate to protect mice in arterial and venous thrombosis models without an increased bleeding risk, the primary complication of all currently used anticoagulants.

## Graphical abstract

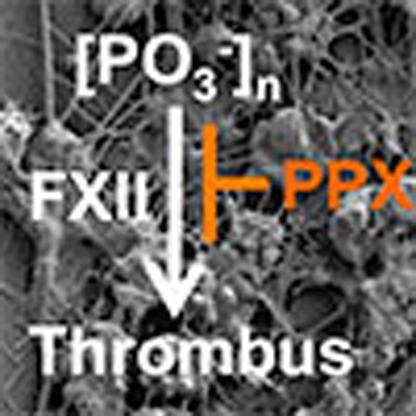

Venous and arterial thromboembolic diseases such as pulmonary embolism, myocardial infarction and stroke are collectively the most common cause of mortality in the developed world^1^. Anticoagulant therapy interferes with the formation of clots within the vasculature and is the mainstay of treatment for the prevention and management of thromboembolic events. Currently, available anticoagulants such as heparin derivatives, vitamin K antagonists (for example, warfarin) and inhibitors of thrombin or factor Xa target enzymes of the coagulation cascade that are critical for fibrin formation^2^. Fibrin constitutes a key component of thrombi. However, it is also required for haemostatic mechanisms that terminate bleeding. Reflecting the dual role of fibrin in thrombosis and haemostasis, increased bleeding is the primary complication of all currently used anticoagulants. This therapy-associated increase in potentially life-threatening haemorrhage partially offsets the benefits of reduced thrombosis^3^,^4^.

Polyphosphate (polyP) is an inorganic polymer of orthophosphate units linked by phosphoanhydride bonds. The polymer is ubiquitously found in all living cells and varies in chain length from just a few to several thousand phosphate units^5^. PolyP functions have been mostly studied in prokaryotes and lower eukaryotes, where polyP contributes to energy metabolism and stress responses as a polymeric storage form of ATP^6^. In mammals, polyP stimulates an array of procoagulant mechanisms and drives fibrin formation. PolyP initiates blood coagulation by activating factor XII (FXII)^7^,^8^,^9^ and amplifies fibrin production by accelerating thrombin-driven feedback activation of factor XI (FXI)^10^ and the conversion of factor V to its active form^8^,^11^. Furthermore, polyP reduces fibrinolysis and enhances the structure of fibrin^12^,^13^. Plasma experiments suggest that the relative potency of polyP in activating these various pathways is dependent on the chain length of the polymer^14^. However, because long-chain polyP is insoluble in the plasma^15^, the relative contribution of polyP to mechanisms of coagulation *in vivo* remains to be established.

*Escherichia coli* exopolyphosphatase (PPX) is a cytoplasmic phosphatase, which catalyses the hydrolysis of intracellular polyP^16^. PPX is composed of four distinct domains^17^, of which the N-terminal domains 1 and 2 harbour the enzymatic activity, whereas the C-terminal domains 3 and 4 mediate substrate binding^18^.

In the present study, we examine recombinant PPX mutants that specifically bind and degrade polyP. Targeting polyP with PPX variants reduces fibrin formation in the plasma, blunts procoagulant activity of activated platelets and interferes with thrombus formation in blood. Neutralizing blood-borne polyP in wild-type (WT) mice protects animals from arterial and venous thrombosis without causing increased bleeding. The anticoagulant effects conferred by targeting polyP are due to interference with FXII activation, and neutralizing polyP does not increase thromboprotection in FXII-deficient (*F12*^−/−^) mice. The data show that polyP operates via FXII *in vivo*, and identify a new strategy for treating thrombosis that involves inhibition of polyP and does not affect haemostasis.

## Results

### PPX deletion mutants

**Figure 1:**
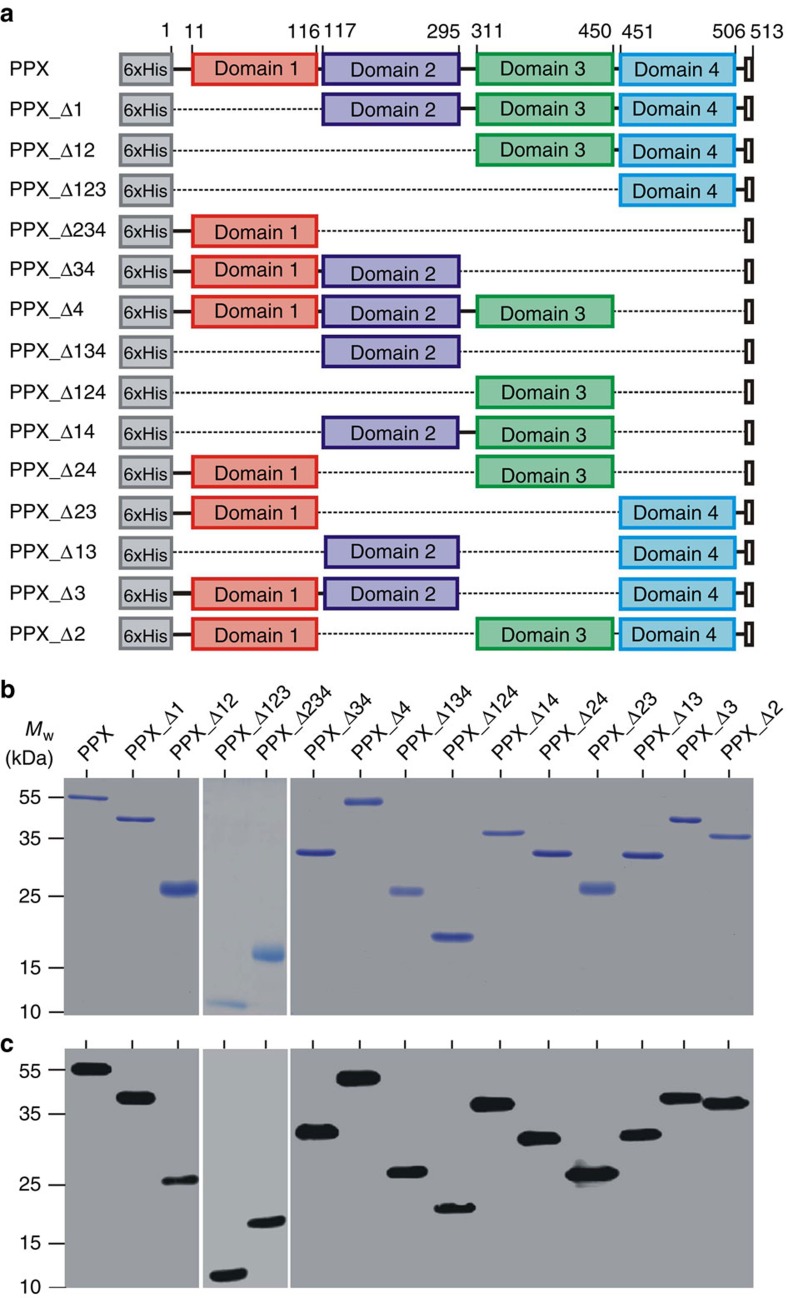
Cloning and expression of PPX mutants. (**a**) Scheme of full-length PPX and PPX deletion mutants lacking various domains. Dark C-terminal squares represent the stop codons, and numbers on top indicate residues. All constructs were fused to an N-terminal 6xHis-tag. Affinity purified proteins were separated by SDS–polyacrylamide gel electrophoresis and visualized by (**b**) Coomassie brilliant blue staining or (**c**) western blotting with an antibody against the 6xHis-tag. A representative photographic film of three independent experiments is shown.

To analyse PPX functionality, we cloned 14 PPX deletion mutants that systematically lack single and combinations of PPX domains using site-directed mutagenesis. A scheme of full-length PPX and PPX mutants created is shown in Fig. 1a. Recombinant *E. coli*-expressed mutants were purified by Ni^2+^-affinity chromatography using their N-terminal 6xHis-tags. All proteins migrated as single bands with an apparent molecular weight that matched their calculated theoretical mass in Coomassie brilliant blue-stained SDS–polyacrylamide gel electrophoresis under reducing conditions (Fig. 1b). Immunoblot analysis confirmed the purity of recombinant proteins (Fig. 1c).

### PPX domains 3 and 4 mediate polyP binding

**Figure 2:**
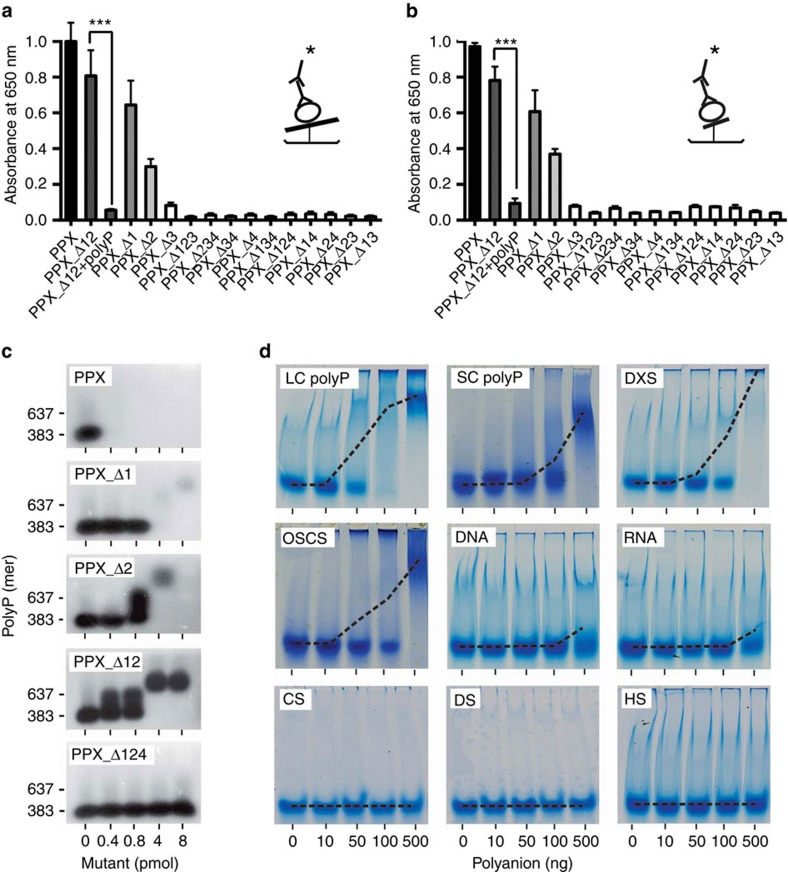
PPX variants binding to polyanions. (**a**,**b**) Immobilized long-chain (LC, **a**) or short-chain polyP (SC, **b**) was incubated for 60 min with 10 nM purified, full-size PPX, PPX deletion mutants or PPX_Δ12 preincubated with polyP. Bound PPX variants were detected using an antibody against the 6xHis-tag, an HRP-coupled secondary antibody and substrate reaction. The cartoon shows the enzyme-linked immunosorbent assay set-up with **——**=LC polyP and **—**=SC polyP, 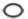
=PPX variant, 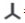
=6xHis-tag antibody and 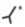
=HRP-coupled detection antibody. Shown are relative amounts of PPX variants binding to polyP. Data blotted relative to full-size PPX, set to 1.0. Mean±s.e.m., *n*=4, ***P<0.001 by Student's *t*-test. (**c**) Gel mobility shift assay of full-size PPX, PPX_Δ1, PPX_Δ2, PPX_Δ12 and PPX_Δ124 binding to polyP. Increasing concentrations of PPX mutants (0–400 nM) were incubated with polyP (7.5 μg ml^−1^) for 30 min, and reaction mixtures containing 0–8 pmol mutant protein per lane were resolved on 1% agarose gels. PolyP was visualized with DAPI-negative staining and synthetic polyP with mean chain length of 383 and 637 phosphate units served as molecular size standard. (**d**) PPX_Δ12 (400 nM) was incubated for 30 min with increasing concentrations of various polyanions including LC polyP, SC polyP, dextran sulfate (DXS), oversulfated chondroitin sulfate (OSCS), DNA, RNA, chondroitin sulfate (CS), dermatan sulfate (DS) and heparan sulfate (HS). PPX_Δ12/polyanion complexes were dissolved on urea-polyacrylamide gels and PPX_Δ12 protein was stained with Coomassie brilliant blue. The dashed line gives PPX_Δ12/polyanion complex formation assessed by densitometric scans. A representative gel of three independent experiments is shown.

We compared PPX mutants for the ability to bind polyP. Long-chain (LC) and short-chain (SC) polyP were covalently coupled to microtiter plates and incubated with PPX mutants, and bound proteins were quantified. EDAC (1-ethyl-3-[3-dimethylamino-propyl]) surface-coupled polyP is protected from PPX degradation^19^. Full-length PPX binding to LC (Fig. 2a; Supplementary Fig. 1A) and SC (Fig. 2b; Supplementary Fig. 1B) polyP was set to 1.0. PolyP binding decreased in mutants PPX_Δ12 (LC: 0.81±0.14; SC: 0.78±0.08), PPX_Δ1 (LC: 0.65±0.14; SC: 0.61±0.12) and PPX_Δ2 (LC: 0.30±0.05; SC: 0.37±0.03). However, PPX mutants lacking domains 3 and 4 alone or in combination did not exhibit polyP binding, and the interaction was reduced to background level (<0.1). Pre-incubating PPX_Δ12 with SC polyP completely inhibited binding to polyP (0.06±0.01 and 0.10±0.03 for LC and SC polyP, respectively), confirming the specificity of mutant PPX for polyP binding.

We confirmed that domains 3 and 4 are required for PPX binding to polyP using a gel shift assay (Fig. 2c). Full-length PPX and mutants PPX_Δ1 and PPX_Δ2 hydrolysed polyP. In contrast, mutant PPX_Δ12 did not degrade polyP, while it still bound to the polymer as indicated by a shift in apparent molecular weight. Further domain deletions led to the loss of polyP binding in mutants PPX_Δ124 (bottom, Fig. 2c) and PPX_Δ123 (Supplementary Table 1). Since PPX_Δ12 presents the minimal construct that bound to polyP but did not degrade the polymer, we used this mutant for further analysis. The interaction between PPX and polyP is mainly driven by charge^17^. We compared PPX_Δ12 binding to polyP with binding to other polyanions (Fig. 2d). Electrophoretic mobility shift assays confirmed dose-dependent interaction of PPX_Δ12 with LC and SC polyP. Similarly, PPX_Δ12 bound to the non-physiologic polyanions dextran sulfate (DXS) and oversulfated chondroitin sulfate that function as FXII contact activators^20^,^21^. In contrast, there was little, if any, binding of PPX_Δ12 to other natural polyanions such as DNA, RNA, chondroitin-, dermatan- or heparan-sulfate.

### PPX specifically degrades polyP

**Figure 3:**
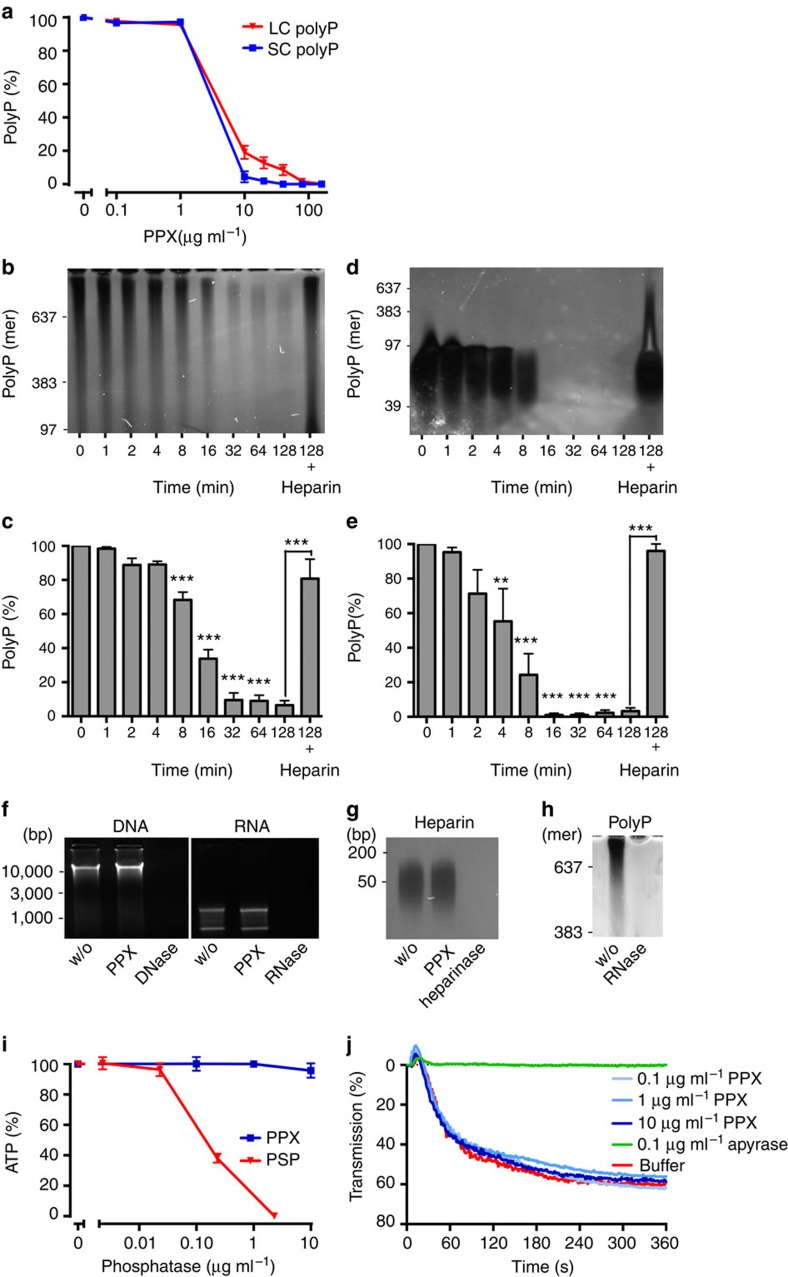
PPX specifically degrades polyP. Concentration- and time-dependent hydrolysis of LC and SC polyP by PPX. (**a**) LC and SC polyP (50 μg ml^−1^ each) were incubated for 30 min with PPX (0–160 μg ml^−1^). Reaction mixtures were separated on 10% urea-polyacrylamide gels, polyP was DAPI-negative stained and signals were blotted relative to buffer-treated polyP (100%). Mean±s.e.m., *n=*4. (**b**,**c**) LC and (**d**,**e**) SC polyP (50 μg ml^−1^ each) were incubated with 10 μg ml^−1^ PPX. Aliquots of 10 μl were taken at indicated time points, resolved on urea-polyacrylamide gels and visualized by negative DAPI staining. Synthetic polyP with mean chain length of 39, 97, 383 and 637 phosphates were loaded as molecular size standard. PolyP incubated with PPX in the presence of the inhibitor heparin (50 μg ml^−1^) is shown in the last lanes. Bars are mean±s.e.m., from four independent experiments, **P<0.01, ***P<0.001 versus 0 min by one-way analysis of variance (ANOVA) and by Student's *t*-test versus heparin addition. (**f**) DNA (1 μg) and RNA (1 μg) were treated with buffer (w/o), PPX (10 μg ml^−1^), DNase (0.1 mg ml^−1^) or RNase (0.5 mg ml^−1^) for 30 min, respectively, and resolved on agarose gels. (**g**,**h**) Heparin (10 μg ml^−1^) or polyP (50 μg ml^−1^) was treated with buffer (w/o), PPX (10 μg ml^−1^), heparinase I (1 U ml^−1^) or RNase (0.5 mg ml^−1^) for 30 min, respectively, separated on urea-polyacrylamide gels and negative DAPI stained. (**i**) ATP (1 μM) was incubated for 30 min with increasing concentrations of PPX (0–10 μg ml^−1^) or shrimp alkaline phosphatase (PSP, 0–2.3 μg ml^−1^) and quantified using a luciferase-based bioluminescence assay. Data are mean±s.e.m., from three independent experiments. (**j**) ADP (5 μM) was treated for 30 min with buffer, apyrase (0.1 U ml^−1^) or increasing concentrations of PPX (0–10 μg ml^−1^). Platelet aggregation in human platelet-rich plasma stimulated by the reaction mixtures. Representative curve of *n=*4.

PPX dose-dependently degraded LC and SC polyP, and 30 min treatment with 10 μg ml^−1^ of the enzyme reduced LC and SC polyP to 19±3% and 4±3%, respectively (Fig. 3a). Incubation of LC and SC polyP with PPX led to time-dependent digestion of the polymer, and after 32 min polyP was reduced to 9±4% (Fig. 3b,c) and 1±1% (Fig. 3d,e), respectively. Addition of the PPX inhibitor heparin (50 μg ml^−1^)^22^ interfered with polyP hydrolysis (81±11% LC and 96±4% SC polyP remaining at 128 min). PPX at a concentration that efficiently degraded polyP (10 μg ml^−1^) did not hydrolyse DNA, RNA or heparin within 30 min, while DNase, RNase and heparinase readily degraded their corresponding substrates (Fig. 3f,g). RNase infusion provides protection from arterial thrombosis in mice^23^. In addition to RNA, RNase (0.5 mg ml^−1^) also degraded polyP within 30 min (Fig. 3h). PPX up to 10 μg ml^−1^ did not significantly reduce ATP levels (96±5%), while 30 min incubation with alkaline phosphatase partially hydrolysed ATP (decrease to 38±3% with 0.2 μg ml^−1^ enzyme; Fig. 3i). PPX also did not interfere with ADP-induced platelet aggregation (60±4% versus buffer: 62±8% transmission; Fig. 3j), indicating that ADP is not a substrate for PPX. In contrast, the ADP hydrolysing enzyme apyrase (0.1 U ml^−1^) completely abolished platelet aggregation (<2% transmission).

### PPX and PPX_Δ12 interfere with polyP-induced coagulation

**Figure 4:**
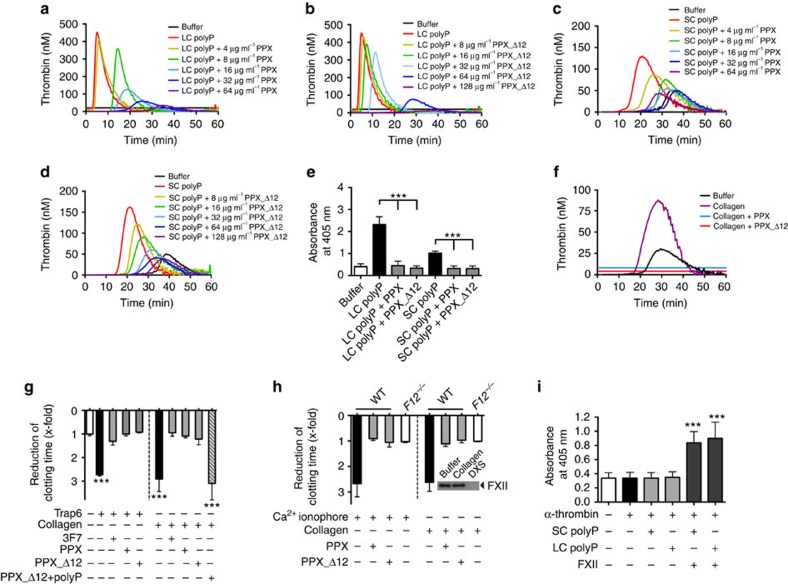
PPX and PPX_Δ12 interfere with polyP-induced coagulation. (**a**,**c**) PPX and (**b**,**d**) PPX_Δ12 inhibit polyP-initiated thrombin formation. Real-time thrombin generation in the absence or presence of increasing concentrations of PPX or PPX_Δ12 in PPP stimulated with long-chain (LC; 1 μg ml^−1^) polyP or short-chain (SC; 10 μg ml^−1^) polyP. Representative thrombin generation curve of *n*=6 is shown. (**e**) FXIIa formation in human plasma was stimulated with buffer, LC (1 μg ml^−1^), SC (10 μg ml^−1^) or LC and SC polyP preincubated with PPX or PPX_Δ12 (100 μg ml^−1^ each). FXIIa was measured by conversion of the chromogenic substrate D–Pro–Phe–Arg–p nitroanilide (S-2302) at *λ*=405 nm and *t*=60 min in the presence of inhibitors specified in the methods. Mean±s.e.m., *n*=6, ***P<0.001 by one-way analysis of variance (ANOVA). (**f**–**h**) Targeting polyP interferes with activated platelet-driven coagulation. (**f**) Real-time thrombin generation in collagen- (3.3 μg ml^−1^) stimulated PRP in the absence or presence of PPX or PPX_Δ12 (500 μg ml^−1^ each). (**g**) Recalcification clotting times in Trap6- (30 μM) or collagen- (33 μg ml^−1^) stimulated human PRP dependent on addition of anti-FXIIa antibody (3F7; 375 nM), PPX, PPX_Δ12 or polyP pre-bound-PPX_Δ12 (500 μg ml^−1^ each). Mean±s.e.m., *n*=4, ***P<0.001 versus buffer by one-way ANOVA. (**h**) Recalcification clotting times triggered by Ca^2+^ ionophore (A23187, 5 μM) or collagen (33 μg ml^−1^) in PRP of WT or *F12*^−*/*−^ mice in the presence (+) or absence (−) of PPX or PPX_Δ12 (500 μg ml^−1^ each). Clotting time reduction is given relative to untreated plasma. Mean±s.e.m., *n*=4. Insert: collagen failed to initiate zymogen FXII activation in PPP, while dextran sulfate (DXS; 100 μg ml^−1^) activated all plasma FXII. (**i**) Role of polyP in FXI activation in the plasma. *F12*^−*/*−^ mouse plasma was incubated with α-thrombin (5 nM) in the absence or presence of polyP (LC or SC, 10 μg ml^−1^ each) and FXII (30 μg ml^−1^). Formed FXIa was measured by conversion of the chromogenic substrate S-2366 at *λ*=405 nm in the presence of inhibitors specified in the methods. Mean±s.e.m., *n*=4, ***P<0.001 versus buffer by one-way ANOVA.

To analyse PPX- and PPX_Δ12-mediated anticoagulant mechanisms, we performed real-time thrombin generation assays in human plasma. Both full-length PPX and PPX_Δ12 dose-dependently reduced total (endogenous thrombin potential) and maximum (peak) thrombin formation and prolonged the lag time and time to peak in plasma stimulated by LC (Fig. 4a,b) or SC (Fig. 4c,d) polyP (Supplementary Table 2). In contrast, PPX and PPX_Δ12 (up to 2 mg ml^−1^ each) did not interfere with thrombin generation induced by the non-physiologic FXII activator silica (Supplementary Fig. 2A–C) or by the activator of the extrinsic coagulation pathway, tissue factor (TF; Supplementary Fig. 2D–G). Similarly, the addition of PPX and PPX_Δ12 had no detectable effect on RNA- or DNA (Supplementary Fig. 2H,I)-induced coagulation. LC and SC polyP stimulated FXIIa production in the plasma (Fig. 4e). Addition of PPX and PPX_Δ12 abolished the ability of polyP to induce FXII zymogen contact activation and resulted in a decrease of FXIIa chromogenic activity. Consistent with previous reports^14^, FXIIa-forming activities of LC polyP exceeded that of SC polyP.

PolyP is released from activated platelets^7^. We analysed PPX and PPX_Δ12 for their ability to interfere with procoagulant platelet activity in human (Fig. 4f,g) and murine (Fig. 4h) platelet-rich plasma (PRP). Collagen-, Trap6- and Ca^2+^ ionophore (A23187)-stimulated platelets initiated thrombin formation, and the addition of PPX and PPX_Δ12 blunted activated platelet-driven coagulation (Fig. 4f; Supplementary Fig. 3A,B). Consistently, platelet stimulation with Trap6 and collagen reduced recalcification clotting times in PRP by 2.7±0.1-fold (71±8 s) and 2.9±0.2-fold (68±9 s), respectively, compared with buffer 1.0±0.1-fold (196±8 s). Addition of PPX and PPX_Δ12 before platelet activation hampered procoagulant platelet activity, and clotting times were close to that of unstimulated controls (PPX: 1.0±0.1/1.1±0.03-fold and PPX_Δ12: 0.9±0.03/1.2±0.1-fold for Trap6 and collagen, respectively). Anti-FXIIa antibody 3F7 (ref. 24) blocked the procoagulant activity of Trap6- or collagen-activated platelets to a similar extent as observed when targeting polyP (1.3±0.2-fold for Trap6 and 1.0±0.1-fold for collagen). PPX_Δ12 that was pre-bound with polyP failed to significantly interfere with procoagulant platelet-mediated clotting (3.1±0.4-fold; Fig. 4g). Consistent with the human data, PPX and PPX_Δ12 abolished the procoagulant activity conferred by collagen- and Ca^2+^ ionophore-stimulated murine platelets (Fig. 4h). The collagen that was used did not activate FXII in the absence of platelets (insert, Fig. 4h). Using purified proteins, polyP was shown to accelerate FXI activation by α-thrombin in a FXII-independent manner^10^. We analysed the relative importance of activated FXI (FXIa) generated by polyP-amplified feedback activation versus FXIa production by polyP-activated FXII (Fig. 4i). Addition of α-thrombin to *F12*^−*/*−^ murine plasma (no detectable FXII antigen by western blotting) triggered FXIa activity, and co-application of LC or SC polyP failed to further amplify FXIa production (LC: 0.35±0.04 and SC: 0.34±0.04 versus 0.34±0.04 for only α-thrombin). In contrast, LC and SC polyP led to significantly higher FXIa activity (LC: 0.90±0.12 and SC: 0.84±0.08) in the plasma containing physiologic amounts of FXII (Fig. 4i).

### PPX and PPX_Δ12 interfere with thrombus formation under flow

**Figure 5:**
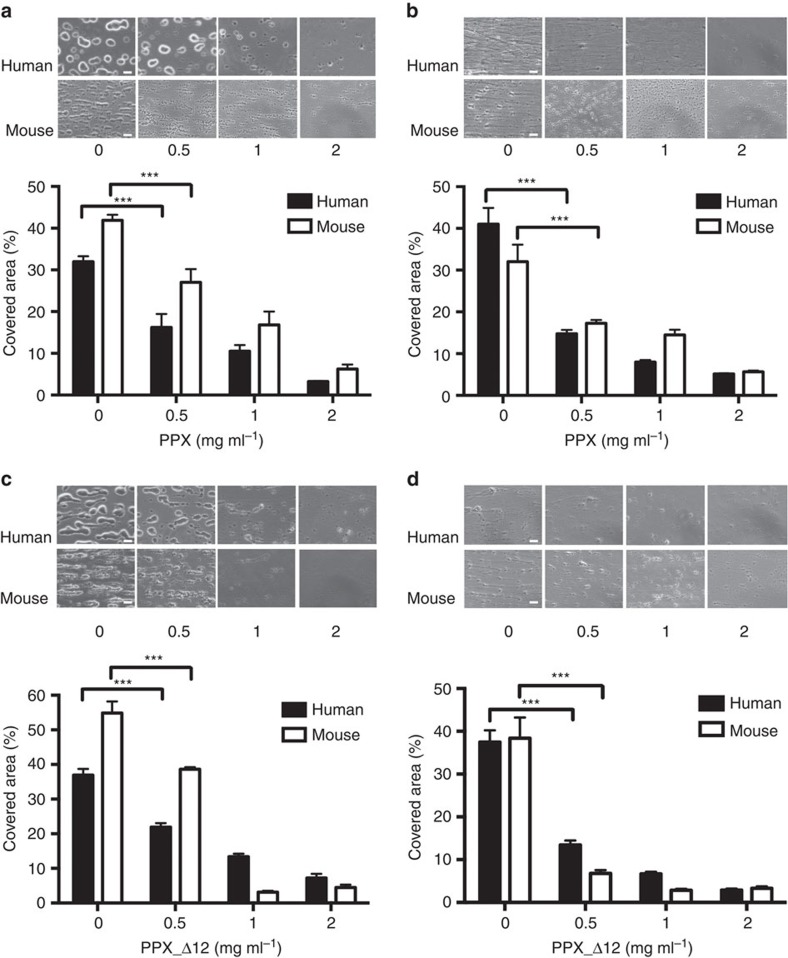
PPX and PPX_Δ12 reduce thrombus formation in blood under flow. Citrated whole blood from human and mice, readjusted to physiologic Ca^2+^ and Mg^2+^ concentrations, was perfused for 4 min over a collagen-coated surface at an arterial (**a**,**c**) or venous (**b**,**d**) shear rate. Representative phase-contrast images of thrombi formed during perfusion in the absence or presence of indicated PPX (**a**,**b**) or PPX_Δ12 (**c**,**d**) concentrations. Scale bars, 20 μm. Columns give the percentage of surface area covered by thrombi. Mean±s.e.m., from four independent experiments, ***P<0.001 by one-way analysis of variance.

**Figure 6:**
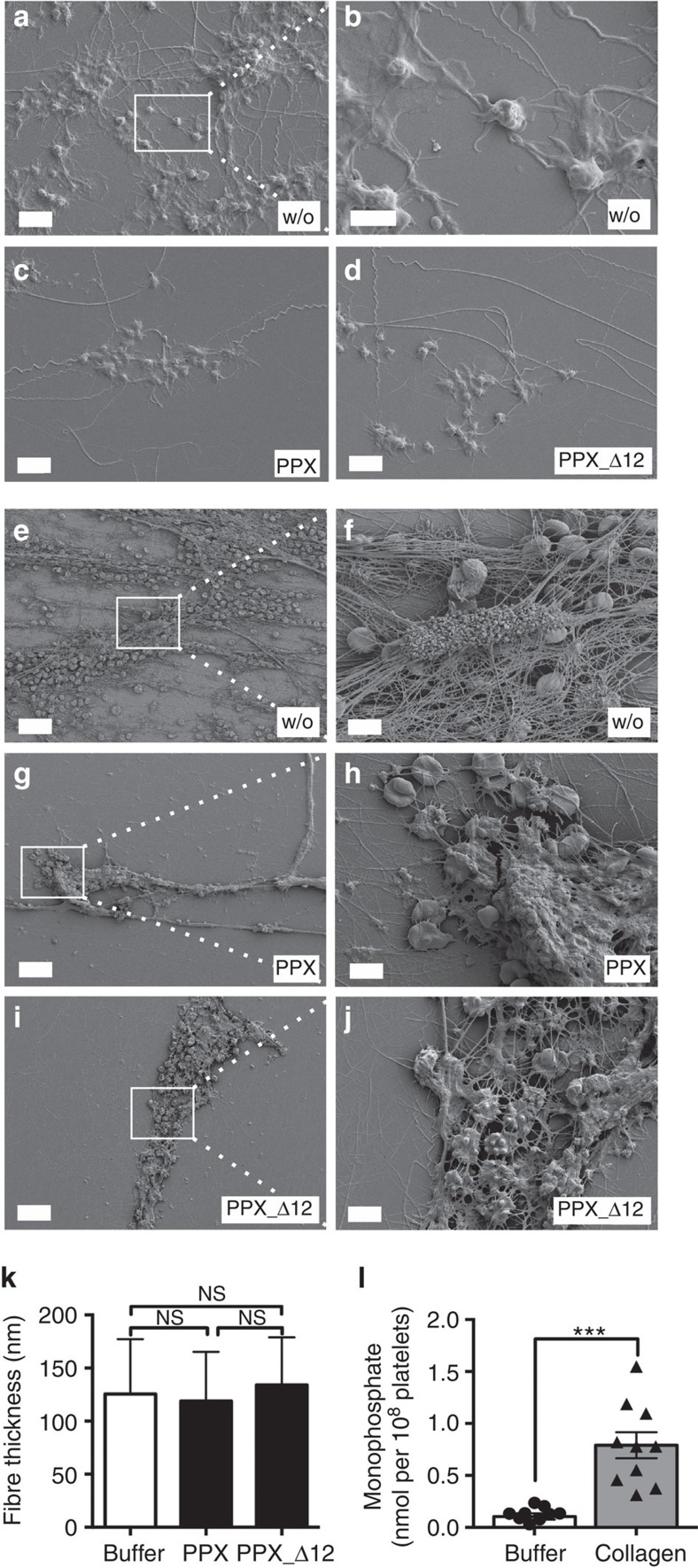
PPX and PPX_Δ12 do not alter fibrin composition. Scanning electron microscopy images of thrombi formed in Fig. 5 under flow. Scanning electron micrographs of thrombi formed in full blood under arterial (**a**–**d**) or venous (**e**–**j**) shear rates in the presence of buffer (w/o), PPX or PPX_Δ12 (1 mg ml^−1^ each). White squares denote the area that is enlarged in **b**, **f**, **h** and **j**). Scale bar, 25 μm (**e**,**g**,**i**); 5 μm (**a**,**c**,**d**,**f**,**h**,**j**); 2 μm (**b**). (**k**) Fibre thickness measured from scanning electron micrographs of 25 fibres in three representative areas. Mean±s.e.m., NS=non-significant by one-way analysis of variance. (**l**) Human platelets were incubated for 20 min with buffer or collagen (10 μg ml^−1^). Released polyP was measured by increase of phosphate in PPX- (50 μg ml^−1^) versus buffer-treated platelet supernatants. Phosphate was calculated from malachite green absorbance at 650 nm from a standard curve. Mean±s.e.m., *n*=10, ***P<0.001 by Student's *t-*test.

Collagen is exposed from the subendothelial matrix at sites of vascular injury. Therefore, we determined whether PPX (Fig. 5a,b) and PPX_Δ12 (Fig. 5c,d) interfered with thrombus formation on collagen-coated surfaces under flow. Citrate-anticoagulated human or murine whole blood was recalcified before being perfused at arterial and venous shear rates of 1,000 s^−1^ (Fig. 5a,c) and 100 s^−1^ (Fig. 5b,d), respectively. In the absence of PPX and PPX_Δ12, platelets initially adhered to collagen fibres and aggregated. Thrombus formation was observed within 4 min from the time of perfusion of human blood (34±2% and 39±3% of surface covered at arterial and venous shear, respectively) and murine blood (48±2% and 35±4% surface covered at arterial and venous shear, respectively). PPX and PPX_Δ12 dose-dependently reduced thrombus formation, and the highest dose (2 mg ml^−1^ each) almost completely abolished thrombosis (<7.5% in human and <6.5% in murine blood). PolyP has been shown to modulate fibrin clot structure in purified systems^12^,^13^. We therefore determined how PPX and PPX_Δ12 affect the composition of thrombi formed in blood under flow. Representative scanning electron microscopy images from thrombi are shown in Fig. 6a–j. Arterial flow conditions resulted in platelet-rich thrombi (Fig. 6a–d), while venous flow rates produced fibrin-rich thrombi (Fig. 6e–j). Under both conditions, the addition of PPX (Fig. 6c,g) and PPX_Δ12 (Fig. 6d,i) slightly reduced platelet deposition but largely diminished the number of fibrin fibres deposited compared with buffer-treated blood (Fig. 6a,e). However, PPX and PPX_Δ12 did not significantly alter the thickness of individual fibrin fibres within the thrombi (126±6 nm versus 119±5 nm and 134±5 nm; Fig. 6k). Finally, we determined how much polyP is secreted from activated platelets. PPX hydrolyses polyP >15 units chain length into orthophosphate^16^. PPX treatment of collagen-stimulated platelet supernatants revealed that 0.79±0.12 nmol polyP (amount expressed as monophosphate) was released into the supernatant from 10^8^ platelets (Fig. 6l).

### Targeting polyP inhibits arterial/venous thrombosis *in vivo*

**Figure 7:**
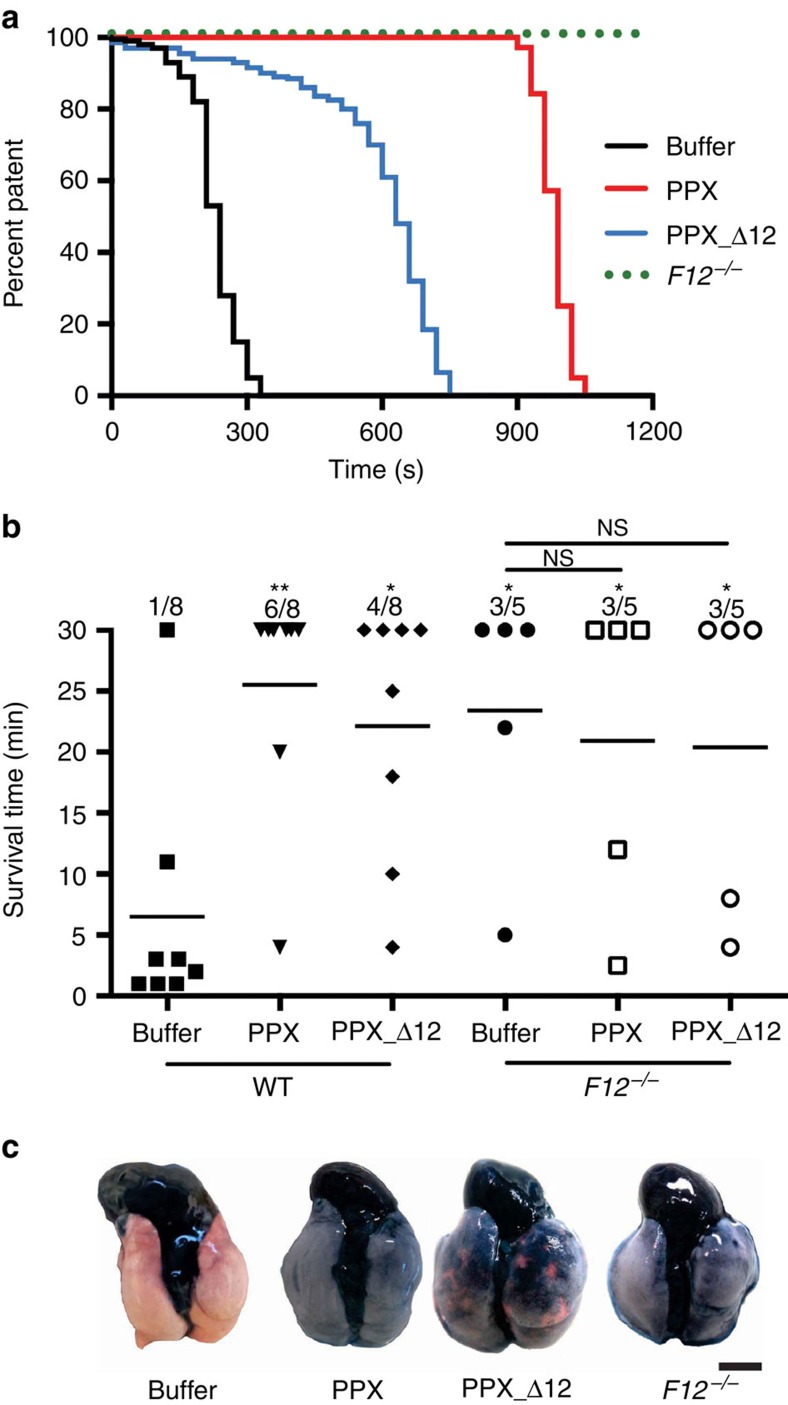
PPX and PPX_Δ12 interfere with arterial and venous thrombosis in mice. (**a**) Thrombosis was induced in the left carotid artery by topical application of 5% FeCl_3_ applied for 3 min in *F12*^−*/*−^ or WT mice that were previously injected with buffer, PPX_Δ12 or PPX (300 mg kg^−1^ BW each). Artery patency was monitored by a flow probe until complete occlusion occurred and zero flow was recorded for >10 min. Representative curves are shown from five independent experiments. (**b**) Pulmonary embolism induced by intravenous infusion of collagen–epinephrine. The survival time of WT or *F12*^−*/*−^ mice pretreated with buffer, PPX or PPX_Δ12 (150 mg kg^−1^ BW each) was monitored. Mortality was assessed in each group of mice, and animals alive 30 min after challenge were considered survivors; **P<0.01, *P<0.05 versus buffer-treated WT by one-way analysis of variance, NS=non-significant. (**c**) Collagen–epinephrine-challenged mice were intravenously infused with Evans blue shortly after the onset of respiratory arrest, while the heart was still beating or after 30 min for those animals that survived. Lungs were excised and perfusion defects were analysed. Occluded parts of the lungs remain their natural pinkish colour. Scale bar, 5 mm.

We next examined our polyP targeting strategy in thrombosis models *in vivo*. We induced thrombosis in the carotid artery by topical application of 5% FeCl_3_ in mice treated with buffer, PPX or PPX_Δ12 (300 mg kg^−1^ body weight (BW) each; Fig. 7a). Vessel occlusion times were significantly prolonged in PPX- and PPX_Δ12-treated mice (PPX: 14.8±2.4 min; PPX_Δ12: 10.4±1.2 min, *n*=5 in each group) over buffer-treated mice (5.9±0.5 min). All (5/5) *F12*^−*/*−^ mice were protected from vessel-occlusive thrombus formation over a 30 min observation period.

We next challenged WT and *F12*^−*/*−^ mice in a model of lethal pulmonary embolism by intravenous infusion of collagen–epinephrine (Fig. 7b). Mice that survived the challenge >30 min were considered survivors. All but one of the buffer-treated WT mice (7/8) died following collagen–epinephrine injection. In contrast, PPX- or PPX_Δ12 (150 mg kg^−1^ BW each)-treated animals were significantly protected from pulmonary thrombosis (6/8 survived for PPX; 4/8 for PPX_Δ12). Consistent with earlier data^25^,^26^, *F12*^−*/*−^ mice were protected from thrombosis in this model (3/5 survived). Infusions of PPX or PPX_Δ12 did not confer an additional survival advantage in *F12*^−*/*−^ mice (3/5 animals survived in each group: buffer, PPX and PPX_Δ12). To confirm pulmonary embolism, we determined lung perfusion by intravenous administration of Evans blue dye (Fig. 7c). Perfused lung areas turned blue, whereas occluded parts remained a natural pinkish colour. Collagen–epinephrine challenge resulted in thrombotic vascular occlusion in buffer-treated mice, visualized by disturbed perfusion of the dye. In contrast, lungs of PPX- or PPX_Δ12-treated WT and *F12*^−*/*−^ mice presented with uniform distribution of the dye, indicating intact vessel perfusion.

### PPX and PPX_Δ12 do not interfere with haemostasis

**Figure 8:**
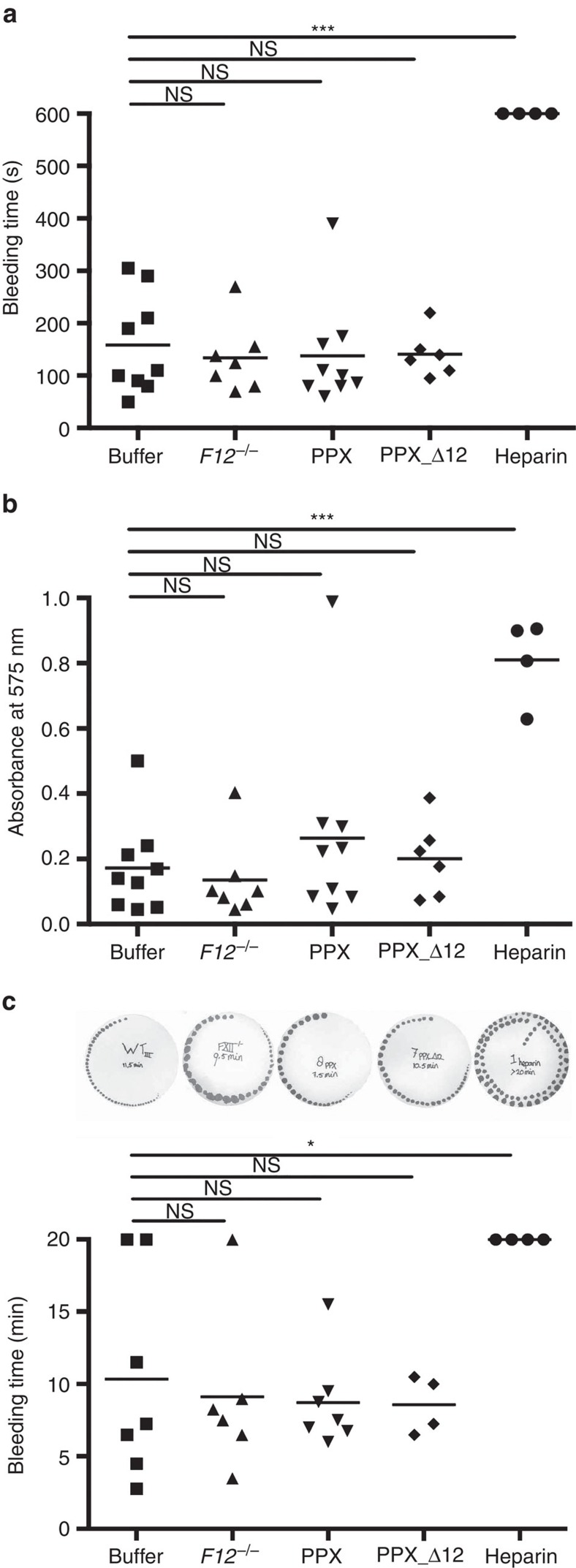
PPX and PPX_Δ12 do not interfere with haemostasis. Wild-type mice were intravenously injected with buffer, PPX, PPX_Δ12 (150 mg kg^−1^ BW each) or heparin (200 U kg^−1^ BW). Bleeding times and blood loss from clipped tail injury assessed the haemostatic capacity of treated and *F12*^−*/*−^ mice. (**a**) Bleeding time and (**b**) total haemoglobin loss determined by absorbance of haemoglobin in 37 °C PBS at *λ*=575 nm. (**c**) Tail-bleeding times were analysed by gently absorbing blood with a filter paper. Each symbol represents one animal; bars within each column indicate the mean, ***P<0.001, *P<0.05, NS=non-significant by one-way analysis of variance.

Bleeding is the major complication of all currently used anticoagulants. We determined the bleeding time and quantified blood loss in buffer, PPX- and PPX_Δ12-treated WT mice challenged by tail clipping. The time to cessation of bleeding of PPX- and PPX_Δ12-treated animals did not significantly differ from control mice (138±34 s and 141±18 s, respectively versus 158±31 s; Fig. 8a). Consistent with previous reports^26^, tail-bleeding times of *F12*^−*/*−^ mice (134±25 s) were similar to those of buffer-treated WT animals. In contrast, heparin-treated (200 U kg^−1^ BW) mice exhibited significantly prolonged bleeding time, uniformly longer than 10 min, which was the observation time of the experiment. The amount of lost haemoglobin as a measure of haemostatic capacity roughly paralleled the bleeding times (Fig. 8b). Infusions of PPX and PPX_Δ12 did not increase blood loss over buffer (absorbance of lost haemoglobin, 0.26±0.10 and 0.20±0.05 for PPX and PPX_Δ12, respectively, versus 0.17±0.05 for buffer). Heparin treatment increased blood loss approximately five times (0.81±0.06) over controls, while FXII deficiency was associated with normal haemostasis (0.18±0.04). In an independent experimental approach, we monitored bleeding from a tail injury site by gently absorbing the blood with a filter paper without touching the wound at 15 s intervals. While haemostasis was impaired in heparin-treated mice (>20 min), bleeding times of PPX-, PPX_Δ12- and buffer-treated mice were similar (8.7±1.2, 8.6±1.0 and 10.4±2.7 min; Fig. 8c).

## Discussion

Fibrin formation constitutes a homeostatic mechanism to prevent excess bleeding; however, fibrin production also contributes to thrombosis^27^. A delicate reaction sequence of clotting factors culminates in fibrin formation. In contrast to all other factors of the coagulation cascade, FXII contributes to thrombosis but appears to be dispensable in haemostasis^28^. Moderate or complete deficiency in FXII is not associated with any haemostatic defects in mice and humans^29^,^30^. On the basis of the unique and selective role of FXII in pathological fibrin production, targeting FXII provides thromboprotection in experimental thrombosis models in mice, rabbits and baboons without affecting the haemostatic capacity of FXII-deficient animals^24^,^31^,^32^,^33^. In the current study, we developed a strategy to specifically interfere with polyP, based on binding and degradation of the polymer. Data show that selective targeting of polyP blocks arterial and venous thrombosis in a FXII-dependent manner without affecting haemostasis.

PolyP initiates FXII contact activation in human plasma^8^, with critical implications for thrombosis *in vivo*^7^. In addition, polyP participates in an array of other procoagulant reactions involving fibrin^12^,^13^, tissue factor pathway inhibitor^8^,^14^, and factors V and XI^11^,^14^,^34^. While polyP has the capacity to modulate these various pathways *ex vivo*, the *in vivo* relevance of the polymer in these mechanisms has remained unknown. When FXII activity was blocked with corn trypsin inhibitor and coagulation was initiated by TF, targeting polyP reduced fibrin deposition and altered clot structure in blood *ex vivo*^35^. Similarly, addition of synthetic polyP (40–367mer) improves clot structure by enhancing fibrin polymerization in a reconstituted system of pure fibrinogen, thrombin and Ca^2+^ (ref. 12). However, in the same system, only slightly shorter polyP (40–174mer) was inactive in increasing fibrin fibre size^13^ and fibrin clot structure^14^. Plasma contains polyP in the submicromolar range^36^. PolyP that was preincubated for 15 min at a concentration over 10 times higher than in plasma was required to increase fibrin fibre thickness^12^. Pyrophosphate abrogates the ability of polyP to enhance fibrin clot structure^14^, and pyrophosphate is found in high concentrations both in plasma and platelet dense granules (∼3 μM (ref. 37) and 326 mM (ref. 38)), arguing against polyP affecting the fibrin structure *in vivo*. Indeed, targeting polyP by PPX and PPX_Δ12 did not alter fibrin composition in whole blood when coagulation was triggered under arterial and venous shear rates (Figs 5 and 6). Defective arterial and venous thrombosis in PPX/PPX_Δ12-infused animals without an associated haemostatic defect (Figs 7 and 8) recapitulates the phenotype of *F12*^−*/*−^ mice^26^. Supporting the notion that polyP affects fibrin formation via FXII, targeting polyP failed to improve protection from thrombosis in *F12*^−*/*−^ mice (Fig. 7). Furthermore, genetic or pharmacologic ablation of FXII abolished polyP-induced thrombosis in rabbit blood^24^ and living mice^7^,^25^,^39^. In contrast to FXII deficiency, low factor V^40^, elevated tissue factor pathway inhibitor^41^ or defective fibrin structure^42^ are associated with increased bleeding. However, targeting polyP does not alter haemostasis nor does it increase blood loss (Fig. 8). This finding argues against a significant contribution of polyP to FXII-independent coagulation mechanisms *in vivo*.

Consistent with the previous data^43^, collagen stimulation of human platelets resulted in the release of ∼1 nmol polyP/10^8^ platelets into the supernatant (Fig. 6). Murine platelets have about half the volume of human platelets and harbour in total ∼30 nmol polyP/10^8^ platelets^44^,^45^. A rough estimate reveals that upon activation, only a small portion of total platelet polyP (<5%) is released into the supernatant. In the plasma, the chain length of disperse synthetic polyP is thought to regulate its activity^9^,^14^. A chromogenic assay indicated that soluble SC polyP has a lower ability to activate FXII compared with LC polymers, leading to the hypothesis that the polymer size regulates polyP activities and that platelet-derived SC polyP is a weak FXII activator^14^. However, *in vivo* regulation of the platelet-derived polymer is probably more complex. Platelets store polyP together with high concentrations of calcium ions in dense granules, and released platelet polyP is complexed with calcium^43^. Calcium-bound polyP has very low solubility and readily precipitates in nanoparticles^46^. The procoagulant properties of polyP packed in nanoparticles largely differ from those of molecularly dissolved molecules. SC polyP in nanoparticle form has significantly higher FXII-activating properties than that of dispersed polyP in solution^15^. The formation of SC polyP aggregates with increased capacity for inducing contact activation argues against a decisive role of polymer chain length in regulating polyP activity *in vivo*. PolyP nanoparticles are stable in physiologic buffers for several hours^15^ and may precipitate on the platelet surface to provide the ideal substrate for efficient FXII contact activation. Similar to these polyP aggregates, SC polyP conjugated with colloidal gold nanoparticles activates FXII with potency equivalent to that of LC polyP^47^. In sum, the data indicate that the localization, composition and functions of platelet polyP are regulated on various levels that warrant further *in vivo* analysis.

An array of studies has demonstrated the contribution of activated FXII to platelet-driven coagulation^48^,^49^,^50^. In support of procoagulant platelets initiating fibrin production by the polyP/FXII pathway, ablation of FXII and platelet polyP impairs thrombosis in murine models^26^,^44^. Furthermore, polyP binding by PPX_Δ12 or degradation by PPX, respectively, almost completely blunted fibrin formation of collagen-, Ca^2+^ ionophore- or Trap6-activated platelets in an FXIIa-dependent manner (Fig. 4). Similar to platelets, polyP and FXII also drive coagulation on the surface of cancer cells and microparticles released from these cells^39^, suggesting that targeting polyP could be a novel therapy in a variety of disease states associated with increased thrombotic risk.

We used PPX and PPX_Δ12 to selectively inhibit polyP (Figs 2 and 3; Supplementary Fig. 2). Previously, recombinant salivary proteins of the African sand fly (PdSP15) were shown to bind polyP and interfere with contact system-driven clotting and inflammation. In addition to polyP, PdSP15 binds to other polyanions and interferes with silica-driven plasma clotting^51^, while PPX_Δ12 binding to polyanions other than polyP is minor (Fig. 2). Cationic polyethylenimine and polyamidoamine dendrimers bind polyP and attenuate thrombosis in murine models^52^,^53^. However, polyamidoamine at a concentration required to provide thromboprotection (8–20 mg kg^−1^ BW) lyses red blood cells^54^ and is cytotoxic to cultured Chinese hamster lung fibroblasts^55^. Alternatively, the crown ether-based universal heparin reversal agents (UHRAs) strongly inhibit polyP procoagulant activity and are non-toxic. In contrast to PPX/PPX_Δ12 (Fig. 4), however, UHRAs also interfered with TF-initiated fibrin formation^56^ that is essential for haemostasis in mice^57^. Indeed, when UHRAs were used in mice at plasma concentrations that reduced thrombosis in the carotid artery, it led to bleeding times approximately threefold higher than controls^56^. In contrast, PPX and PPX_Δ12, even at the highest concentrations tested (2 mg ml^−1^), selectively targeted polyP and did not interfere with nucleic acid-, silica- and TF-induced coagulation (Supplementary Fig. 2). Supporting the notion that polyP mostly functions via FXII activation *in vivo*, our strategies for specific neutralization of polyP interfered with thrombosis but did not impair the haemostatic capacity of treated animals, reproducing the thromboprotective phenotype of FXII-deficient animals (Fig. 8). Unlike PPX-based strategies, UHRAs increase bleeding. The mechanisms of UHRAs' interference with haemostatic mechanisms are not precisely known and, similarly to protamine (a basic protein clinically used as a heparin antidote), may involve charge-driven interference with factor V activation^58^. Extracellular RNA has been considered to promote thrombosis *in vivo* by inducing FXII contact activation. This notion is based on the fact that infusion of RNase (an enzyme that degrades RNA) interferes with arterial thrombosis in a murine FeCl_3_-driven vascular injury model^23^. However, RNase also readily hydrolyses polyP (Fig. 3), offering an alternative explanation for the thromboprotective effects conferred by the enzyme.

In this study, we are leveraging the identification of a new pathway of thrombosis treatment that involves the inhibition of polyP with phosphatases and does not affect haemostasis. Currently used anticoagulant drugs target individual or a combination of coagulation factors downstream in the coagulation cascade and are therefore subject to significant bleeding risk. In contrast, PPX interferes with the initiation of the coagulation process *in vivo*. PPX has the potency to degrade polyP of various sizes and structures, which represents a therapeutic advantage, especially in platelet-rich arterial thrombi where the local polyP concentration might be high^59^. FXIIa formation and contact system-driven coagulation are critical initiators of thrombosis when blood is exposed to artificial non-physiologic surfaces such as medical device-associated thrombosis^60^, extracorporeal membrane oxygenation^24^ and dialysis membranes^61^. Importantly, FXIIa levels are elevated in patients following cardiac stenting^62^. In support of a role for FXII activation by bio-incompatible surfaces, an active site-directed anti-FXIIa antibody prevents thrombin activation and fibrin formation as effectively as heparin in rabbits on an extracorporeal membrane oxygenation system, without altering haemostasis^24^. Development of materials with low propensity to cause contact activation would reduce the risk of device-associated thrombosis^63^. Similar to nanoparticle drug-eluting stents coupled with a direct thrombin inhibitor^64^, targeting procoagulant polyP by PPX immobilized on catheters, stents or artificial heart valves could provide a safe novel approach of anticoagulation that can be employed in FXII-related disease states. Furthermore, the enzymatic activity of PPX would facilitate degradation of multiple polyP molecules, potentially exceeding the antithrombotic activity of currently used anticoagulants such as thrombin and FXa inhibitors that target a single coagulation protease. Overall, this study demonstrates that polyP degradation by PPX represents a new modality for treating thrombosis without increasing bleeding risk.

## Methods

### Cloning of PPX deletion mutants

Genomic DNA of *E. coli* (XL 10-gold, Stratagene) was extracted with phenol/chloroform. The following primers were used to clone PPX (L06129.1): 5′-CAC**CTCGAG**AATGCCAATACACGAT-3′ and 5′-**GAATTC**CCCGCAAAGTATTAAAGCGG-3′ (start and stop codons are underlined; restriction sites are in bold). XhoI and EcoRI restriction sites were used to insert the amplified DNA fragment into the pTrcHisB expression vector (Invitrogen) leading to pTrcHisB-PPX. Mutants based on the PPX domain organization were cloned by PCR-based mutagenesis using the QuikChange Site-Directed Mutagenesis kit (Stratagene) and pTrcHisB-PPX as template. To generate PPX_Δ1, PPX_Δ12 and PPX_Δ123, primers *ppx-XhoI12* (5′-GGTCATTCCCTACCCGATTGAAATTAT**CTCGAG**TAATGAAGAAGCCCGT-3′), *ppx-XhoI23* (5′-AACTGCGCCTTTCTGACGG**CTCGAG**TCGCGAAGGCGTACTGTATG-3′) or *ppx-XhoI34* (5′-AATCAACGTCAGGCAAC**CTCGAG**TCCGCCAACATTGACGCTG-3′; new restrictions sites are indicated in bold) mutated nucleotides 348–353, 885–890 and 1,356–1,361 of the PPX cDNA, into XhoI restriction sites. XhoI digest and re-ligation of mutated pTrcHisB-PPX vectors produced PPX deletion mutants. For PPX mutants PPX_Δ234, PPX_Δ34 and PPX_Δ4, nucleotides 339–344, 909–914 or 1,326–1,331 in the PPX cDNA, respectively, were mutated to EcoRI sites using primers: *ppx-EcoRI12* (5′-GTCATTCCCTACCCGATT**GAATTC**ATTTCCGGTAATGAAGAAGC-3′), *ppx-EcoRI23* (5′-CGAAGGCGTACTGTAT**GAATTC**GAAGGACGTTTCCGTCAT-3′) or *ppx-EcoRI34* (5′-AGCTATTGCGCCTTGGC**GAATTC**CTCAACAATCAACGTCAG-3′). PPX_Δ124 and PPX_Δ14 were produced from PPX_Δ12 and PPX_Δ1 coding vectors, respectively, using primer *ppx-EcoRI34*. PPX_Δ134 was generated using PPX_Δ34 coding vector with primer *ppx-XhoI12*. To create PPX_Δ23, PPX_Δ13 and PPX_Δ3, the sequence coding for domain 4 (derived from EcoRI digestion of *ppx-EcoRI34* mutated pTrcHisB-PPX) was cloned into the EcoRI site of PPX_Δ234, PPX_Δ134 and PPX_Δ34 coding vectors, respectively. For PPX_Δ24 and PPX_Δ2, nucleotides 369–374 and 903–908 in PPX_Δ4 and PPX coding vectors, respectively, were mutated into PstI sites, using: *ppx-PstI12* (5′-GGTAATGAAGAAGCCCGT**CTGCAG**TTTATGGGCGTGGAACATACC-3′) and *ppx-PstI23* (5′-GTTACGCGAAGGCGTA**CTGCAG**GAAATGGAAGGACGTTTCC-3′). The mutated products were PstI digested and re-ligated. DNA sequencing confirmed all plasmids.

### Expression and purification of PPX deletion mutants

Ultracompetent *E. coli* were transformed with pTrcHisB vectors coding the various PPX deletion mutants. Recombinant protein expression was induced by 0.5 mM isopropylthio-β-D-galactoside (Sigma-Aldrich) at 37 °C for 4 h. Bacteria were collected by centrifugation, resuspended in binding buffer (20 mM NaH_2_PO_4_, 500 mM NaCl and 20 mM imidazole, pH 7.4) and lysed by sonication. Cell lysates were centrifuged (10,000 *g* for 10 min at 4 °C) and supernatants were loaded on 1 ml HisTrap FF crude column (GE Healthcare). Following washing, bound proteins were eluted with 20 mM NaH_2_PO_4_, 500 mM NaCl and 500 mM imidazole, pH 7.4. Fractions containing mutants were combined, and solvent was changed to PBS, pH 7.4, using desalting columns (Econo-Pac 10 DG, Bio-Rad). Protein concentrations were determined by the Bradford method. Coomassie brilliant blue staining assessed protein purity. Western blotting was performed using 6xHis-tag antibody (1:1,000, Merck Millipore, catalogue number #70796-3) and horseradish peroxidase (HRP)-coupled anti-mouse Fc antibody (1:5,000, Jackson ImmunoResearch, #115-035-003). Full scans of the western blots are shown in Supplementary Fig. 4.

### Binding of mutants to microplate-immobilized polyP

LC (ILC Performance Products) polyP with chain lengths of 200–1,000 monomers and SC polyP with chain lengths of 0–400 monomers were Ca^2+^-preadsorbed and immobilized onto high-binding polystyrene 96-well plates (Immune 2 HB, Thermo Scientific) using EDAC carbodiimide-mediated covalent coupling^19^. In brief, wells were incubated with polyethylenimine (400 ng ml^−1^) in 0.1 M sodium carbonate–bicarbonate buffer, pH 9.2, overnight at 37 °C. Thereafter, wells were incubated with 200 μl of LC or SC polyP solution (25 μg ml^−1^ polyP in 50 mM EDAC, 77 mM 2-[N-morpholino] ethanesulfonic acid hydrate and 1 mM CaCl_2_, pH 6) for 4 h. Unbound polyP was removed by 2 × washing with 2 M LiCl followed by 2 × washing with water. Plates were blocked for 2 h with 5% gelatin in PBS, pH 7.4. Full-length PPX and PPX deletion mutants (200 μl, 10 nM each, corresponding to 0.3 μg ml^−1^ for PPX_Δ12) were incubated for 1 h at 37 °C in PBS supplemented with 0.6% gelatin and 0.05% Tween. Bound PPX mutants were detected using 6 × His-tag antibody (2 μg ml^−1^), HRP-coupled detection antibodies (1:5,000) and substrate reaction (3,3′,5,5′-tetramethylbenzidine; Sigma-Aldrich) at an absorbance wavelength of 650 nm.

### Gel shift assays

PolyP (7.5 μg ml^−1^) with a defined chain length of 383 phosphate units was incubated with increasing concentrations (0–400 nM) of full-length PPX or PPX deletion mutant for 30 min at 37 °C. Reaction mixtures containing 0–8 pmol mutant protein per lane were resolved on 1% agarose gels in TBE (89 mM Tris, 89 mM boric acid, 2 mM EDTA, pH 8.3), and polyP was visualized using 4,6-diamidino-2-phenylindole (DAPI)-negative staining^14^. To compare PPX_Δ12 binding to different polyanions, PPX_Δ12 (400 nM) was incubated for 30 min at 37 °C with increasing concentrations (0–50 μg ml^−1^) of polyP, oversulfated chondroitin sulfate (SERVA electrophoresis), DXS (Sigma-Aldrich), DNA, RNA, chondroitin sulfate (Sigma-Aldrich), dermatan sulfate (Sigma-Aldrich) and heparan sulfate (Sigma-Aldrich). Reaction mixtures containing 0–500 ng polyanion per lane were resolved on 6% polyacrylamide TBE-urea (7 M) gels, and PPX_Δ12 was visualized using Coomassie brilliant blue staining. The maximum of PPX_Δ12/polyanion complex signal intensity in stained gels was determined from densitometric scans using ImageJ 1.37 software and was blotted relative to PPX_Δ12 protein signal intensity in the absence of polyanion.

### Polyphosphate degradation assays

LC and SC polyP (50 μg ml^−1^) were incubated with increasing concentrations (0–160 μg ml^−1^) of PPX for different times (0–128 min). In some experiments, heparin (50 μg ml^−1^) was added to inhibit PPX enzymatic activity. All reaction mixtures were separated on 10% polyacrylamide TBE-urea (7 M) gels, and polyP was visualized with DAPI-negative staining. PolyP samples with defined chain length were used as size standards^14^. Relative amounts of polyP were determined by densitometric scans using ImageJ software.

DNA and RNA (1 μg) were treated with PPX (10 μg ml^−1^), DNase (0.1 mg ml^−1^, Sigma-Aldrich) or RNase (0.5 mg ml^−1^, Qiagen), and were analysed on 1% agarose gels containing Gel Red Nucleic Acid Stain (Biotum). Heparin (10 μg ml^−1^, Sigma-Aldrich) was incubated with PPX or heparinase I (1 U ml^−1^, Sigma-Aldrich), analysed on a 10% polyacrylamide TBE-urea (7 M) and visualized with negative DAPI technique. ATP (1 μM) was treated with PPX (0–10 μg ml^−1^) or shrimp alkaline phosphatase (0–2.3 μg ml^−1^, Roche), and ATP was quantified using a luciferase-based bioluminescence assay according to the instructions from the ATP determination kit (Invitrogen).

### Blood collection

Human plasma was obtained from healthy volunteers with informed consent. Sampling at the Karolinska University Hospital was approved by the Stockholm ethical committee (Regionala Etikprövningnämden). Peripheral venous blood was collected into 3.2% trisodium citrate (9:1 blood-to-citrate ratio). The first 10 ml of sample was discarded. Platelet-poor plasma (PPP) was prepared by two consecutive centrifugation steps at 3,000 *g* for 10 min each. PRP was prepared by centrifugation at 250 *g* for 10 min. Platelets were counted, and the platelet count was adjusted to 250 × 10^9^ l^−1^ with autologous PPP. PRP preparation lead to minute thrombin formation^65^. Plasma from individuals with inherited FXII and FXI deficiency was obtained from George King Bio-Medical, Inc. Factor levels were below the western blot detection limit.

### Animals

All animal care and experimental procedures were performed at Karolinska Institutet, complied with the principles of laboratory and animal care and were approved by the ethical committee of Stockholm's Norra Djurförsöksetiska Nämnd. *F12*^−*/*−^ mice are backcrossed for >10 generations to *C57BL/6* background^7^. Male and female mice of 6–12 weeks of age without blinding or randomization were used. For blood collection, mice were anaesthetized by intraperitoneal injection of 2,2,2-tribromoethanol and 2-methyl-2-butanol and subjected to retro-orbital blood sampling. Blood was collected into 3.8% trisodium citrate, and PRP was obtained by differential centrifugation of the citrated blood at 100 *g* for 10 min followed by 60 *g* for 8 min.

### Platelet aggregation

Human PRP (450 μl, 250 × 10^9^ platelets per litre) was incubated for 2 min at 37 °C in a two-channel aggregometer (Chrono-log corporation) before platelets were stimulated with ADP (5 μM, Sigma-Aldrich). ADP was preincubated with PPX (0–10 μg ml^−1^) or apyrase (0.1 U ml^−1^, Sigma-Aldrich) for 30 min at 37 °C. Change in light transmission during constant stirring of the samples was recorded for 6 min and normalized to light transmission of PPP.

### Real-time thrombin generation

Thrombin formation in real time was analysed with the calibrated automated thrombography method using a Fluoroscan Ascent fluorometer (Thermo Scientific) equipped with a dispenser (Thrombinoscope BV) as previously described^24^. In brief, coagulation was stimulated by LC polyP (1 μg ml^−1^), SC polyP (10 μg ml^−1^), RNA (5 μg ml^−1^, isolated from human colon cancer cells by the Qiagen RNeasy mini kit according to manufacturer's instructions), DNA (5 μg ml^−1^, Sigma), TF (1 or 5 pM) or with a commercial silica activator (STA-PTT-Automate 5, according to the manufacturer's instructions) in 60 μl PPP to a total volume of 120 μl containing 4 μM phospholipids (Thrombinoscope BV), 16.6 mM Ca^2+^ and 2.5 mM fluorogenic substrate (ZGGR-AMC, Thrombinoscope BV). PolyP, TF or PTT Automate 5 were preincubated for 30 min at 37 °C with PPX or PPX_Δ12 (0–2 mg ml^−1^ each) before being added to the plasma. Thrombin formation in PPX- or PPX_Δ12 (500 μg ml^−1^ each)-supplemented PRP on stimulation with Horm's collagen (3.3 μg ml^−1^, Takeda), Trap6 (30 μM, Bachem) or Ca^2+^ ionophore (A23187, 5 μM, Sigma-Aldrich) was performed as detailed^54^. Thrombin formation was quantified using the Thrombinoscope software package (Version 3.0.0.29).

### FXIIa amidolytic activity assays

LC polyP (1 μg ml^−1^) or SC polyP (10 μg ml^−1^) was preincubated with PPX or PPX_Δ12 (100 μg ml^−1^ each) for 30 min at 37 °C and added to pooled normal human plasma. Although S-2302 is commonly used to determine FXIIa activity, the chromogenic substrate can be cleaved by plasma kallikrein, thrombin and FXIa with rates 7 × higher than, equivalent to and 60 × lower than, respectively, compared with FXIIa^66^. To overcome this limitation, we analysed S-2302 cleavage in the plasma in the presence of the plasma kallikrein inhibitor DX-88 (10 μM, Ecallatide, Dyax), FXI antibody (15 μg ml^−1^, XI1)^67^ and thrombin inhibitor hirudin (10 U ml^−1^, Sigma-Aldrich). Alternatively, the plasma from individuals with inherited FXI deficiency (George King Bio-Medical Inc.) was spiked with DX-88 and hirudin. Formed FXIIa was analysed using the chromogenic substrate S-2302 (1 mM, Chromogenix) at an absorbance wavelength of 405 nm in a Bio-Kinetics Reader (SpectraMax Plus, Molecular Devices) at 37 °C.

### Recalcification time

Recalcification times were measured using a Kugel-koagulometer (ABW Medizin und Technik GmbH) in 50 μl human or murine (WT or *F12*^−*/*−^) PRP. Human PRP was preincubated with Trap6 (30 μM), Horm's collagen (33 μg ml^−1^) or buffer and murine PRP was preincubated with Ca^2+^ ionophore (A23187, 5 μM), collagen (33 μg ml^−1^) or buffer at 37 °C for 10 min in the absence or presence of freshly purified PPX, PPX_Δ12 (500 μg ml^−1^ each) or FXIIa inhibitory antibody (3F7, CSL Limited, 375 nM). Clotting was initiated by recalcification with 50 μl of a 25 mM CaCl_2_ solution in a final volume of 150 μl.

### FXII activation assay *in vitro*

FXII activation was analysed as previously described^68^ with some minor modifications. Human citrated PPP was activated with high-molecular-weight DXS (100 μg ml^−1^, 500 kDa, Sigma-Aldrich), collagen (33 μg ml^−1^) or buffer. Samples were incubated for 30 min at 37 °C. Reducing Laemmli sample buffer was added to stop the reactions, and the samples were boiled for 5 min followed SDS–polyacrylamide gel electrophoresis on 10% gels. Western blotting was performed using primary anti-FXII antibody (1:1,000, Nordic Immunological Laboratories, GAHu/FXII) and HRP-coupled secondary antibody (1:5,000, Jackson ImmunoResearch, #205-032-176). Full scan of the western blot is shown in Supplementary Fig. 4C.

### FXIa amidolytic activity assay

Plasma from *F12*^−*/*−^ mice was incubated for 30 min at 37 °C with α-thrombin (5 nM, Haematologic Technologies Inc), LC or SC polyP (10 μg ml^−1^ each) and Gly-Pro-Arg-Pro peptide, which inhibits fibrin polymerization (1 mM, Sigma-Aldrich). Some samples were reconstituted with human FXII (30 μg ml^−1^, Molecular Innovations). The chromogenic substrate S-2366 (1 mM, Chromogenix) can also be cleaved by thrombin, plasma kallikrein, FXa and FXIIa, with rates 1.5 × higher and 2 × , 20 × and 35 × lower, respectively, than that of FXIa^66^. Therefore, after incubation CTI (100 μg ml^−1^, Haematologic Technologies Inc), DX-88 (10 μM), Rivaroxaban (0.5 μM, Bayer Pharma AG) and hirudin (10 U ml^−1^) were added to neutralize FXIIa, plasma kallikrein, FXa and thrombin, respectively. Formed FXIa was analysed using the chromogenic substrate conversion at an absorbance wavelength of 405 nm.

### Thrombus formation under flow

Thrombus formation under flow was analysed as previously described^24^ with some minor modifications. Coverslips were coated with collagen (0.5 μg applied to 10 mm^2^) and blocked with HEPES buffer (136 mM NaCl, 2.7 mM KCl, 0.42 mM NaH_2_PO_4_, 5 mM Hepes, 2 mM MgCl_2_ and 1% BSA, pH 7.4) for 30 min. Coverslips were placed onto a transparent, 50 μm-deep parallel-plate flow chamber (Maastricht Instruments BV), which was pre-rinsed with BSA-containing buffer. Chambers were co-infused with citrate-anticoagulated blood and isotonic CaCl_2_/MgCl_2_ solution (10:1 ratio for human blood, 1:1 ratio for murine blood) by pulse-free pumps, which resulted in free Ca^2+^ and Mg^2+^ concentrations of ∼2 mM each. Blood samples were preincubated for 30 min with increasing concentrations of freshly purified PPX or PPX_Δ12 (0–2 mg ml^−1^). After 4 min of flow (shear rates of 100 s^−1^ for venous or 1,000 s^−1^ for arterial flow conditions), flow chambers were rinsed with HEPES buffer (pH 7.4) containing 2 mM CaCl_2_. Phase-contrast images were recorded on an ORCA-Flash 2.8 CMOS Camera (Hamamatsu) and Nikon Eclipse Ti microscope equipped with × 20 objective. Surface covered areas were assessed by the analysis of images using Image 4.0 software.

### Electron microscopy

Electron microscopy was performed as described previously^69^. In brief, thrombi formed in human blood spiked with buffer, PPX or PPX_Δ12 (1 mg ml^−1^ each) under flow were fixed by immersion in 2.5% glutaraldehyde in 0.1 M phosphate buffer, pH 7.4. Specimens were rinsed with distilled water and placed at room temperature in 70% ethanol for 10 min, 95% ethanol for 10 min, absolute ethanol for 15 min and pure acetone for 10 min, and then transferred to tetramethylsilane (Merck) for 10 min and air dried. After drying, thrombi were mounted on an aluminium stub and coated with platinum (Bal-Tec SCD 005). The thrombi were analysed in an Ultra 55 (Carl Zeiss) field emission scanning electron microscope at 3 kV. The thickness of fibrin fibres was measured in each of three representative areas using GraphicConverter for a total of 25 fibres per thrombi.

### PolyP analysis in platelet supernatants

Washed platelets were incubated for 20 min with Horm's type collagen (10 μg ml^−1^) or prostaglandin E1 (5 μM, Sigma-Aldrich) in HEPES-Tyrode's buffer. Supernatants were incubated for 2 h with buffer or PPX (50 μg ml^−1^) at 37 °C. Orthophosphate in the supernatants was determined by the malachite green-based Phosphate Assay Kit (Colorimetric; ab65622, Abcam). Increase of phosphate in PPX- versus buffer-treated supernatants indicates released polyP^44^. Phosphate was calculated from absorbance at 650 nm from a standard curve. All polyP concentrations in this experiment are given as concentration of phosphate monomers^14^.

### Arterial thrombosis model

FeCl_3_-induced arterial thrombosis was performed as described previously^26^,^70^ with minor modifications. In brief, mice were anaesthetized with intraperitoneal injection of 2,2,2-tribromoethanol and 2-methyl-2-butanol (0.5 g kg^−1^ BW). A segment of the carotid artery was exposed, and a flow probe size 0.5 (Transonic) was inserted around the artery to monitor blood flow. Thrombus formation was induced by topically applying a piece of filter paper (1 × 1.5 mm) saturated with 5% FeCl_3_. After 3 min, the filter paper was removed, and the blood flow was continuously recorded until the flow rate was 0 ml min^−1^ for 10 min. Mice were injected intravenously with buffer only or freshly purified PPX_Δ12 or PPX (300 mg kg^−1^ BW each) 10 min before challenge. One animal died during surgery before FeCl_3_ challenge and was excluded.

### Pulmonary thromboembolism model

Mice were anaesthetized by intraperitoneal injection of 2,2,2-tribromoethanol and 2-methyl-2-butanol (0.5 g kg^−1^ BW) and intravenously injected with buffer only or freshly purified PPX_Δ12 or PPX (150 mg kg^−1^ BW each) 10 min before challenge. Horm's collagen (200 μg kg^−1^ BW) was mixed with epinephrine (60 μg kg^−1^ BW, Sigma-Aldrich) and slowly injected into the inferior vena cava. Animals surviving the challenge >30 min were considered survivors. After the onset of respiratory arrest and while the heart was still beating or after 30 min for those animals that survived, Evans blue dye (1% in 0.9% saline) was intravenously infused^39^. Lungs were excised and photographed. Two anaesthetized animals with unstable breathing before collagen–epinephrine injection were excluded.

### Tail-bleeding assay

Bleeding times were determined as previously described^26^. Mice were anaesthetized and injected intravenously with buffer, 150 mg kg^−1^ BW freshly purified PPX_Δ12 or PPX or 200 U kg^−1^ BW heparin. After 10 min, the mouse tail was transected 3 mm from the tip with a razor blade. The bleeding tail was immersed in a 15 ml test tube containing 12 ml pre-warmed PBS. Bleeding time was recorded as the time to cessation of bleeding for 10 s. Blood loss was quantificated by measuring the haemoglobin content of blood collected into PBS. Following centrifugation, the pellet was lysed with lysis buffer (8.3 g l^−1^ NH_4_Cl, 1.0 g l^−1^ KHCO_3_ and 0.037 g l^−1^ EDTA) and the absorbance of the sample was measured at 575 nm. In addition, tail-bleeding times were analysed with a filter paper dabbed to the wound at 15 s interval without disrupting the forming clot. The experiment was continued until bleeding stopped completely or at 20 min.

### Statistical methods

Sample or experiment sizes were determined empirically; no statistical tests were used to predetermine the size of the experiments. To check whether the data were normally distributed, a quantile–quantile plot was used and data were analysed by Student's *t*-test or, in the case of multiple comparisons, one-way analysis of variance followed by *post hoc* analysis using Tukey's multiple comparisons test. Prism 6.0 (Graph Pad) was used for analysis, and the values of probability *P*<0.05 were considered as statistically significant. *In vitro* and *in vivo* data are expressed as mean values±s.e.m., unless otherwise indicated.

### Data availability

The authors declare that the data supporting the findings of this study are available within the article and from the authors on request.

## Additional information

**How to cite this article:** Labberton, L. *et al*. Neutralizing blood-borne polyphosphate *in vivo* provides safe thromboprotection. *Nat. Commun.* 7:12616 doi: 10.1038/ncomms12616 (2016).

## Supplementary Material

Supplementary Information Supplementary Figures 1-4 and Supplementary Tables 1-2.
