# Supplementary material for: Neutralizing blood-borne polyphosphate in vivo provides safe thromboprotection
Source: Nat Commun. 2016 Sep 6;7:12616. doi: 10.1038/ncomms12616 (PMC5025862; doi:10.1038/ncomms12616)
Supplement: Supplementary Information — Supplementary Figures 1-4 and Supplementary Tables 1-2. [file ncomms12616-s1.pdf]

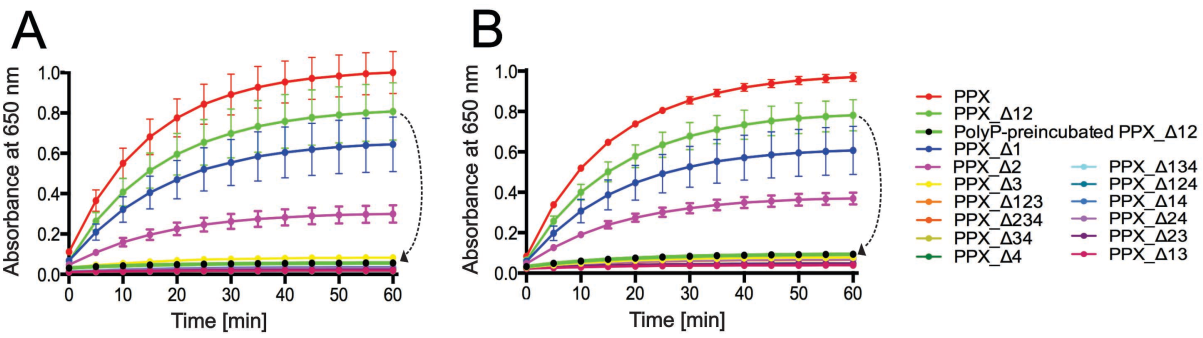

SUPPLEMENTARY FIGURE 1. PPX variants binding to polyP.

Immobilized long-chain (A) or short-chain polyP (B) was incubated for 60 min with 10 nM purified full-size PPX, PPX deletion mutants or PPX_Δ12 pre-incubated with polyP. Bound PPX variants were detected using an antibody against the 6xHis-tag, an HRP-coupled secondary antibody and substrate reaction. Kinetics of PPX variants binding to polyP is shown. Data blotted relative to full-size PPX, set to 1.0. Mean ± SEM, *n*=4.

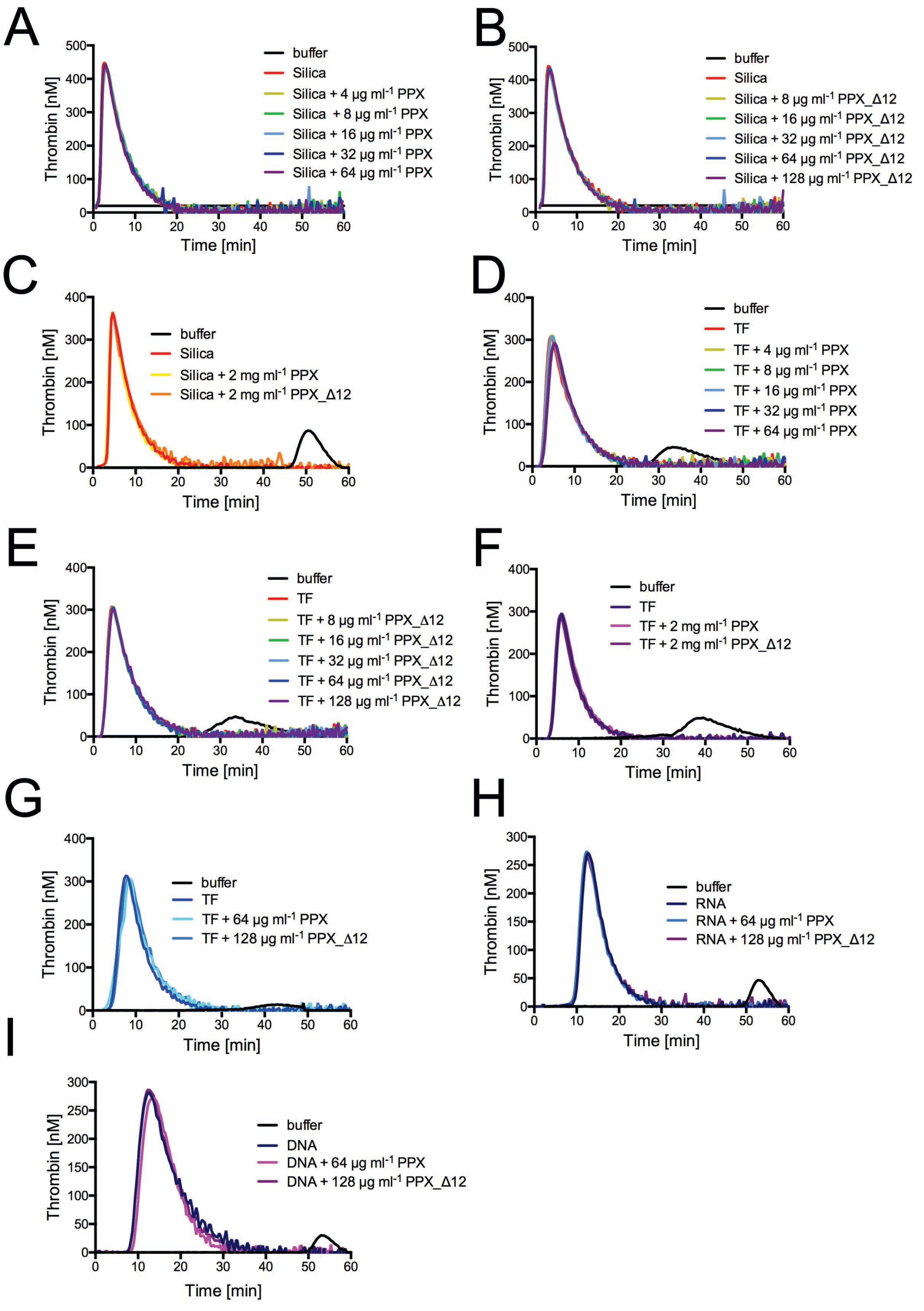

SUPPLEMENTARY FIGURE 2. PPX and PPX_Δ12 do not interfere with silica-, TF-, RNA- or DNA-induced thrombin formation.

Real-time thrombin generation in the absence or presence of increasing concentrations of PPX or PPX_Δ12 in PPP stimulated with silica (A-C), TF (5 pM, D-F; 1 pM, G), RNA (5 µg ml^{-1}, H) or DNA (5 µg ml^{-1}, I). Representative curves of a series of *n*=4 are shown.

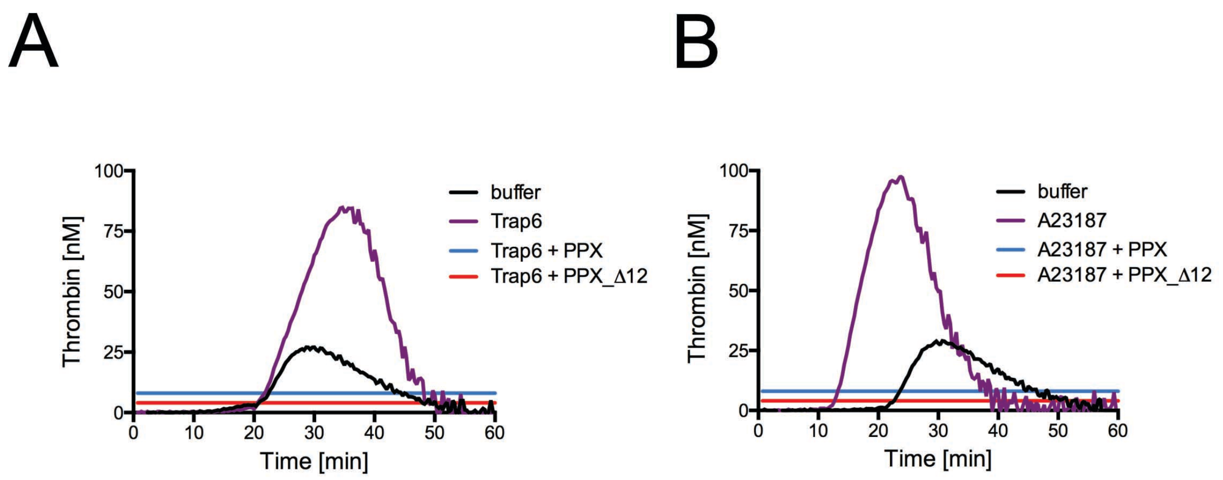

SUPPLEMENTARY FIGURE 3. PPX and PPX_Δ12 reduce activated platelet-driven thrombin generation.

Real-time thrombin generation in PRP stimulated with (A) Trap6 (30 μM) or (B) Ca^{2+} ionophore (A23187, 5 μM) in the absence or presence of PPX or PPX_Δ12 (500 μg ml^{-1} each). Representative curves from 4 independent experiments are shown.

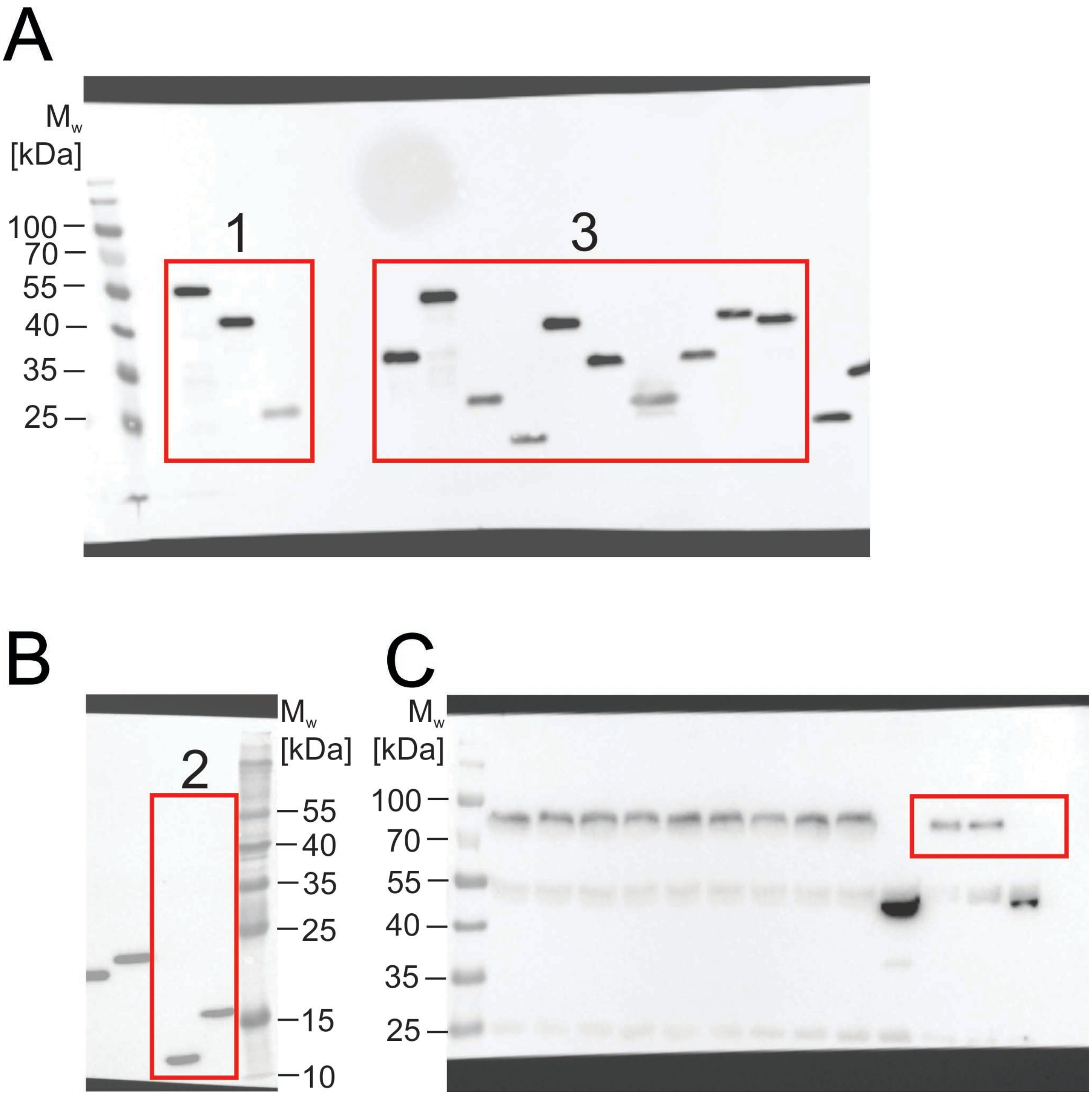

SUPPLEMENTARY FIGURE 4. Full scans of Western blots.

(A, B) Uncropped scans of Western blots are shown in manuscript figure 1C. Red squares denote areas that are depicted in the manuscript. Square 2 is inserted between squares 1 and 3. (C) Uncropped scan of the insert in manuscript figure 4H.

SUPPLEMENTARY TABLE 1: *Polyphosphate binding and degradation capacity of PPX mutants.*

Mutant	Binding	Degradation
PPX	+	+
PPX_Δ1	+	+
PPX_Δ12	+	-
PPX_Δ123	-	-
PPX_Δ234	-	-
PPX_Δ34	-	-
PPX_Δ4	-	-
PPX_Δ134	-	-
PPX_Δ124	-	-
PPX_Δ14	-	-
PPX_Δ24	-	-
PPX_Δ23	-	-
PPX_Δ13	-	-
PPX_Δ3	+	+
PPX_Δ2	+	+

SUPPLEMENTARY TABLE 2: *Effects of PPX and PPX_Δ12 on real time thrombin generation.*

	**Lag time (min)**	**Time to peak (min)**	**Peak thrombin (nM)**	**ETP (nM*min)**
**LC polyP**	3.8 ± 0.3	5.8 ± 0.4	418 ± 21	2727 ± 87
+ PPX	27.3 ± 1.6	35.1 ± 0.1	34 ± 1	450 ± 20
+ PPX_Δ12	24.6 ± 1.2	38.4 ± 0.6	18 ± 1	281 ± 17
**SC polyP**	16.1 ± 5.3	21.3 ± 0.2	175 ± 84	1748 ± 291
+ PPX	24.8 ± 3.1	30.5 ± 2.7	32 ± 16	454 ± 197
+ PPX_Δ12	32.1 ± 3.4	39.7 ± 3.4	33 ± 7	435 ± 117

LC and SC polyP-induced thrombin formation in the presence of PPX (64 μg ml^{-1}) and PPX_Δ12 (128 μg ml^{-1}). Values are expressed as mean ± SEM, *n*=6. ETP=endogenous thrombin potential.
